# Declining COVID-19 morbidity and case fatality in Germany: the pandemic end?

**DOI:** 10.1007/s15010-022-01873-0

**Published:** 2022-06-18

**Authors:** Ulf Dennler, Fabian Geisler, Christoph D. Spinner

**Affiliations:** 1 Staff Office Strategic Medical Controlling, University Hospital Ulm, Faculty of Medicine, University of Ulm, Albert-Einstein-Allee 29, 89081 Ulm, Germany; 2 Department of Internal Medicine II, University Hospital Rechts der Isar, Technical University of Munich, School of Medicine, Ismaninger Str.22, 81675 Munich, Germany

The Severe Acute Respiratory Syndrome Coronavirus 2 (SARS-CoV-2) caused the 2019 global Coronavirus disease (COVID-19) pandemic. A high disease burden, increasing hospitalization rates, and daily mortality numbers were reported in many countries during the initial waves of the pandemic. In the absence of specific prevention and therapy, non-pharmaceutical public health interventions targeted social distancing, contact reduction and universal masking to contain infections [1]. Subsequently, different COVID-19 vaccines became licensed, resulting in effective prevention of infection and disease [2]. Shortly after the global vaccines became available, reports showed waning vaccine efficacy, necessitating a booster vaccination [3]. Furthermore, novel variants challenged the effectiveness of the vaccines. The Omicron variant was described first in November 2021 and was simultaneously associated with an increased likelihood of transmission but reduced morbidity and mortality in vaccinated patients [4, 5]. As a result, it remains unclear whether the infection prevention strategy is still adequate to reduce morbidity and mortality.

Publicly available data on reported infections, deaths, and vaccinations were analyzed to study this question in the German population (eRef 1). Infections and vaccinations were recorded cumulatively as a proportion over time. Case fatality (CF) over time was calculated from the 7-day average data for infections and deaths. This was compared with the cumulative course for the first, second, or booster vaccination and infection rates.

Ultimately, the German vaccination campaign was initiated focusing on vulnerable persons and healthcare workers (HCWs) on December 27, 2020. Within the following 8 months, 60% of the population received at least one vaccination, while a CF reduction from 4.5% to approximately 0.5% was observed. While from September to November 2021, an increase in CF to 1% was observed, from September 2021 onwards, a national booster campaign was carried out, focusing on vulnerable persons at risk of severe COVID-19 and the HCWs.

Between mid and end of December 2021, the Omicron spread increased to 70%, while at the same time, CF decreased from 0.75% to 0.5%. With Omicron infections rising rapidly thereafter, CF dropped to 0.1% simultaneously (Fig. 1).

**Fig. 1:**
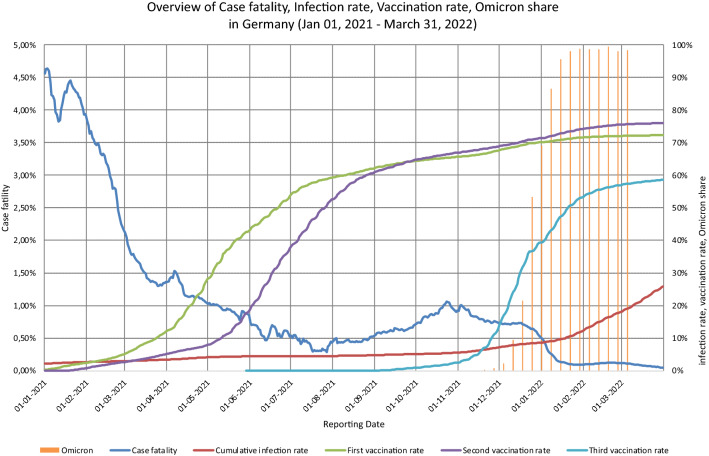
Covid-19 case fatality based on 7-day average of cases and deaths; cumulative rates of vaccinations, infections, and Omicron share in Germany (complete data sources of this herein presented generated graph: eRef 1)

In conclusion, parallel to the initial vaccination campaign CF dropped by 90% in the pre-Omicron era. Very likely associated with increased immune competence of more than 80% by vaccinations or convalescence and the parallel emergence of Omicron, CF and vaccination rates became independent in twice vaccinated or convalesced people. Public health measures should be carefully re-evaluated and focus on morbidity and mortality instead solely infection prevention.

## Supplementary Information

Below is the link to the electronic supplementary material.

Supplementary file1 (DOCX 15 kb)
