# Supplementary material for: Declining COVID-19 morbidity and case fatality in Germany: the pandemic end?
Source: Infection. 2022 Jun 18;50(6):1625–6. doi: 10.1007/s15010-022-01873-0 (PMC9206462; doi:10.1007/s15010-022-01873-0)
Supplement: Supplementary file 1 — Supplementary file1 (DOCX 15 kb) [file 15010_2022_1873_MOESM1_ESM.docx]

**eAppendix:**

**eRef 1: Publicly available data Sources for by authors generated Figure 1**

Data for infections and deaths until 03/31/2022: Robert Koch-Institut (RKI), dl-de/by-2-0 (<https://www.arcgis.com/home/item.html?id=f10774f1c63e40168479a1feb6c7ca74>); retrieved on 10-04-2022

Data for vaccinations until 31-03-2022: Robert Koch-Institut (2021): COVID-19-Impfungen in Deutschland, Berlin: Zenodo. DOI:[10.5281/zenodo.5126652](http://doi.org/10.5281/zenodo.5126652) (<https://github.com/robert-koch-institut/COVID-19-Impfungen_in_Deutschland/blob/master/Aktuell_Deutschland_Landkreise_COVID-19-Impfungen.csv>); retrieved on 10-04-2022

German residents 2020: Statistisches Bundesamt, (<https://www.destatis.de/DE/Themen/Laender-Regionen/Regionales/Gemeindeverzeichnis/Administrativ/Archiv/GVAuszugJ/31122020_Auszug_GV.xlsx?__blob=publicationFile>); retrieved on 10-04-2022
